# Human Lung Spheroids as In Vitro Niches of Lung Progenitor Cells with Distinctive Paracrine and Plasticity Properties

**DOI:** 10.5966/sctm.2015-0374

**Published:** 2016-09-22

**Authors:** Isotta Chimenti, Francesca Pagano, Francesco Angelini, Camilla Siciliano, Giorgio Mangino, Vittorio Picchio, Elena De Falco, Mariangela Peruzzi, Roberto Carnevale, Mohsen Ibrahim, Giuseppe Biondi‐Zoccai, Elisa Messina, Giacomo Frati

**Affiliations:** ^1^ Department of Medical‐Surgical Sciences and Biotechnology, “Sapienza” University of Rome, Rome, Italy; ^2^ Department of Medical‐Surgical Science and Translational Medicine, “La Sapienza” University of Rome, Rome, Italy; ^3^ Department of AngioCardioNeurology, Istituto di Ricovero e Cura a Carattere Scientifico Neuromed, Pozzilli, Italy; ^4^ Department of Pediatrics and Neuropsychiatry, “Umberto I” Hospital, Rome, Italy

**Keywords:** Lung stem cells, Pneumospheres, Three‐dimensional culture, Stem cell niche, Epithelial‐to‐mesenchymal transition

## Abstract

Basic and translational research on lung biology has discovered multiple progenitor cell types, specialized or facultative, responsible for turnover, renewal, and repair. Isolation of populations of resident lung progenitor cells (LPCs) has been described by multiple protocols, and some have been successfully applied to healthy human lung tissue. We aimed at understanding how different cell culture conditions may affect, in vitro, the phenotype of LPCs to create an ideal niche‐like microenvironment. The influence of different substrates (i.e., fibronectin, gelatin, laminin) and the impact of a three‐dimensional/two‐dimensional (3D/2D) culture switch on the biology of LPCs isolated as lung spheroids (LSs) from normal adult human lung biopsy specimens were investigated. We applied a spheroid culture system as the selective/inductive step for progenitor cell culture, as described in many biological systems. The data showed a niche‐like proepithelial microenvironment inside the LS, highly sensitive to the 3D culture system and significantly affecting the phenotype of adult LPCs more than culture substrate. LSs favor epithelial phenotypes and LPC maintenance and contain cells more responsive to specific commitment stimuli than 2D monolayer cultures, while secreting a distinctive set of paracrine factors. We have shown for the first time, to our knowledge, how culture as 3D LSs can affect LPC epithelial phenotype and produce strong paracrine signals with a distinctive secretomic profile compared with 2D monolayer conditions. These findings suggest novel approaches to maintain ex vivo LPCs for basic and translational studies. Stem Cells Translational Medicine *2017;6:767–777*

**Significance Statement**

A lung niche‐like microenvironment was created ex vivo inside the pneumosphere. Pneumospheres have a distinctive secretomic profile compared with two‐dimensional culture. Three‐dimensional culture switch significantly affects lung progenitor phenotype. Lung progenitors in pneumospheres were more committed toward epithelial lineages.

## Introduction

Respiratory diseases are among the leading causes of morbidity and mortality worldwide, with lung infections, lung cancer, and chronic obstructive pulmonary disease together accounting for almost 10 million deaths worldwide during 2008 [1, 2]. Hospitalization and health care costs due to lung diseases represent a great burden, and the proportion of morbidity and mortality is expected to remain stable in future years [2]. Lung transplantation is an effective option for several end‐stage lung diseases. It remains available only for a restricted number of patients, however, and is associated, nevertheless, with several implications.

The biology of airway stem cell compartments in the adult has become a rapidly advancing field. Multiple progenitor populations have been identified as either specialized or facultative, depending on the conditions analyzed, such as physiological turnover or response to injuries [3, 4]. It is already well established that the lung is a very plastic organ, with many examples of dedifferentiation, transdifferentiation, and cell cycle reentry to replenish lost parenchymal cell pools, leading to a model of multiple (and sometimes interchangeable) stemness compartments, depending on the nature and the extent of injury [5, 5, 6, 7, 8].

Isolation of different mesenchymal or epithelial populations of resident lung progenitor cells (LPCs) has been described by multiple protocols and criteria, with only few of them having been successfully applied to healthy human lung tissue [9, 10, 11, 12, 13, 14], and with remaining uncertainty about the best markers or subpopulations to be used for clinical translation. Such ex vivo models are necessary not only for regenerative medicine purposes but can also be intended for multiple research needs, such as pharmacological studies or modeling of lung tissue and diseases, and, more recently, for ex vivo lung bioengineering protocols using decellularized whole lungs as scaffolds [15].

The biological complexity of the lung requires careful consideration of the conditions used to mimic ex vivo the ideal microenvironment for LPCs. Phenotype control of isolated cells represents a delicate task to manage in vitro, for both basic and translational research. Multiple stimuli play synergic roles on lung stem cell niches, modulating proliferation, commitment, epithelial‐to‐mesenchymal (EMT) or the opposite mesenchymal‐to‐epithelial transition, and crosstalk with stroma [4, 9, 16, 17, 18, 19, 20]. The extracellular matrix has a central influence as well. Different proteins are known to affect progenitor phenotype and regenerative potential, such as laminin [21, 22], fibronectin [21, 23], and collagen/gelatin [24], depending on the cell type and on the developmental stage. Moreover, standard two‐dimensional (2D) culture systems deeply differ from the in situ microenvironment, whereas three‐dimensional (3D) conditions often provide multiple advantages for many systems [25, 26, 27].

Thus, it is mandatory to understand how different cell culture variables, such as substrate or a 3D system, may affect in vitro the phenotype of LPCs so an ideal microenvironment for their isolation can be created that could be readily adjusted for modulating their commitment and phenotype. In this study, we have investigated the influence of different substrates and a 3D/2D culture switch on the biology of LPCs isolated as spheroids from normal adult human lung biopsy specimens, whose translational relevance has been recently highlighted [14], characterizing their differential phenotype and paracrine properties. We show how culture as spheroids can significantly increase LPCs’ epithelial commitment compared with culture as monolayers, and how they can gain paracrine and phenotypical features of an ex vivo epithelial progenitor cell niche. Our data support for lung tissue the same notion derived from other tissues; that is, in vitro 3D culture systems are among the most effective approaches to control stemness/commitment balance, and to create ex vivo a functional niche‐like microenvironment for progenitor maintenance and commitment.

## Materials and Methods

### Sample Collection, Processing, and Cell Cultures

**Figure 1:**
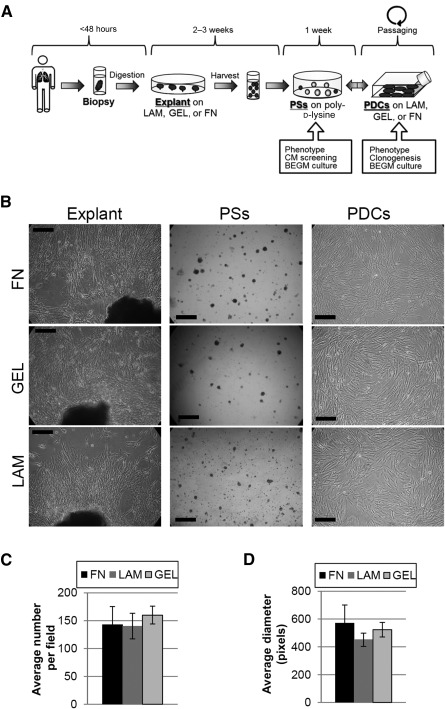
Culture protocol and experimental timing of pneumosphere explant culture. **(A):** Schematic representation of cell culture protocol. **(B):** Representative phase‐contrast images of lung explant cultures, PSs, and PDCs on different coatings. Yield was analyzed by averaging the PS number per field **(C)** and the PS diameter **(D)**, and was similar on the three coatings tested. Scale bars = 250 µm. Abbreviations: BEGM, bronchial/epithelial growth medium; CM, conditioned medium; FN, fibronectin; GEL, gelatin; LAM, laminin; PDC, pneumosphere‐derived cell; PS, pneumosphere.

The study was approved by the Ethics Committee of the “Sant'Andrea” Hospital, “La Sapienza” University (Protocol no. 5195/2013). After institutional review board approval and signed informed consent, lung tissue was obtained from 3 patients (1 26‐year‐old woman and 2 men, ages 18 and 20 years) who underwent lung surgery for benign disease at the Division of Thoracic Surgery, “Sant’Andrea” Hospital (“La Sapienza” University of Rome, Italy). Lung tissue biopsies were obtained during video‐assisted thoracoscopic surgery procedures using a mechanical stapler (Echelon Endopath; Johnson & Johnson, Somerville NJ, http://www.ethicon.com). Cell culture protocol was based on other spheroid culture systems [9, 10, 14, 28, 29]. In particular, lung tissue from each patient was thoroughly washed in phosphate‐buffered saline (PBS) and mechanically minced into 1‐ to 2‐mm^3^ pieces, digested for 15 minutes in 0.05% trypsin‐EDTA, divided into equal amounts, and then plated as primary explants on Petri dishes coated with fibronectin (FN; BD Biosciences, East Rutherford NJ, http://www.bdbiosciences.com), gelatin (GEL), or laminin (LAM; Sigma‐Aldrich, St. Louis MO, http://www.sigmaaldrich.com) in complete explant medium (CEM). CEM medium is made of Iscove’s modified Dulbecco’s medium (IMDM) supplemented with 20% fetal bovine serum (FBS), 1% penicillin‐streptomycin, 1% l‐glutamine, and 0.1 mM 2‐mercaptoethanol. After 3–4 weeks, outgrowth explant‐derived cells (EDCs) were harvested by serial washes with PBS, EDTA, and mild trypsinization, and plated on poly‐d‐lysine‐coated multiwell plates at 6,000 cells per cm^2^ in pneumosphere growth medium (PGM). PGM medium is made of 35% IMDM plus 65% Dulbecco’s modified Eagle’s medium (DMEM)/F‐12 mix, supplemented with 3.5% serum, 1% penicillin‐streptomycin, 1% l‐glutamine, 0.1 mM 2‐mercaptoethanol, 1 unit/ml thrombin (Sigma‐Aldrich), 1:50 B‐27 (Thermo Fisher Scientific Life Sciences, Waltham, MA, http://www.thermofisher.com), 80 ng/ml basic fibroblast growth factor, and 25 ng/ml epidermal growth factor (EGF; Peprotech, Rocky Hill, NJ, http://www.peprotech.com). After 1 week, lung spheroids, here named pneumospheres (PSs), were collected by pipetting and centrifugation (50 relative centrifugal force [rcf]), and plated as PS‐derived cells (PDCs) on the corresponding coating in CEM media. Secondary PSs (IIPSs) were obtained again by replating PDCs on poly‐d‐lysine coating. For differentiation stimuli, PDCs and PSs were cultured for 1 week in bronchial/epithelial growth medium (BEGM; Lonza, Basel, Switzerland, http://www.lonza.com). The isolation procedure is summarized in Figure 1A. Spheroid dissociation was achieved by 10‐minute incubation in trypsin 0.05% with gentle pipetting every 2 minutes.

### Resident Lung Mesenchymal Stem Cell isolation and Transdifferentiation Assays

Lung mesenchymal stem cells (L‐MSCs) were isolated from three lung biopsy specimens that were collected as reported above and as previously described [30, 31]. Briefly, tissue was processed by enzymatic digestion in 1 mg/ml collagenase type‐I (Thermo Fisher) and 0.05% trypsin/0.02% EDTA (Biowest, Nuaille, France, www/biowest.net) for 45 minutes. Digested tissue was then filtered with 70‐µm cell strainers, centrifuged, and plated in low‐glucose DMEM supplemented with 20% FBS. Transdifferentiation assays were performed on EDCs, PDCs, and L‐MSCs by culture for 14 days in StemPro Chondrogenesis (2.5 × 10^5^ cells per cm^2^), Adipogenesis (1 × 10^4^ cells/cm^2^) and Osteogenesis (5 × 10^3^ cells per cm^2^) Differentiation kits (all Thermo Fisher). Cells were then fixed and stained with Alcian Blue, Oil Red O solution, or Alizarin Red (Sigma‐Aldrich), respectively, according to the manufacturer instructions, as previously described [30, 31].

### Clonogenesis and Cell Proliferation and Yield

For clonogenesis experiments, PDCs were cultivated at very low density (5 cells per cm^2^ in 100‐mm Petri dishes) for 2 weeks, then fixed and Giemsa stained (Sigma‐Aldrich). Dishes were imaged and semiautomatic colony counting was performed with ImageJ software (National Institutes of Health, Bethesda, MD, https://imagej.nih.gov/ij) for cloning efficiency calculation. Clusters of cells with a diameter >5 mm were considered as clones. PSs yield was calculated 1 week after seeding by semiautomatic particle count performed by ImageJ software on at least 8 random fields per condition. PSs dimension was calculated by ImageJ measurements of the average diameter on at least 20 random PSs per condition.

### Cytofluorimetry

Cells were collected by trypsinization and stained for 30 minutes with the following primary antibodies: CD105, CD44, CD45, CD117, CD133 (Abcam, Cambridge, MA, http://www.abcam.com), and CD31 (Sigma‐Aldrich). Cells were then incubated for 15 minutes with anti‐rabbit or anti‐mouse Alexafluor‐488 (Thermo Fisher) secondary antibodies. Also, directly conjugated PE‐CD326/EpCam (Biolegend, San Diego, CA, www.biolegend.com) and FITC‐CD90 (Dianova, Hamburg, Germany, http://www.dianova.com) antibodies were used, together with 7AAD labeling (BD Biosciences) to exclude dead cells. Appropriate isotype‐matched immunoglobulins were used as controls, and appropriate preliminary experiments were performed for compensation settings. A total of 10,000 events were recorded with a FACSAria II cytometer (BD Bioscience) using DiVa Software (version 6.1.1; BD Biosciences). Analysis was performed using Flowing software (version 2.5.1; Turku Centre for Biotechnologies, Turku, Finland, http://www.btk.fi).

### RNA Extraction and Real‐Time Polymerase Chain Reaction

RNA was extracted with a column‐based kit (Qiagen, Valencia, CA, https://www.qiagen.com). Reverse transcription was performed on a 500‐ng starting total of RNA (Thermo Fisher) in a 20‐µl reaction, and complementary DNA product was then subjected to real‐time polymerase chain reaction (PCR) with Sybr Green Mix in a 7900HT Fast Real‐Time system (Thermo Fisher). All primer sets were previously tested for optimal efficiency and all reactions were analyzed at the end by melting curves to confirm product specificity. Each reaction was performed in triplicate. The comparative cycle threshold method was used for relative quantification, using 18S as the housekeeping gene; reference conditions used for normalization are specified in each data set. Supplemental online Table 1 lists primer sequences.

### Immunofluorescence

Cells were fixed for 10 minutes with 4% paraformaldehyde at 4°C, permeabilized with 0.1% Triton X‐100 (Sigma‐Aldrich) in PBS with 1% bovine serum albumin, then blocked in 10% goat serum, incubated overnight at 4°C in 1% goat serum with primary antibodies, and then incubated for 2 hours at room temperature with Alexa‐conjugated secondary antibodies (Thermo Fisher) and 4′,6‐diamidino‐2‐phenylindole nuclear dye (Sigma‐Aldrich). Primary antibodies were ki67, Oct4, TTF1, cytokeratin 5 (KRT5), prosurfactant‐C (pro‐SFTPC), aquaporin 5 (AQP5; all Abcam), vimentin (VIM), cytokeratin 18 (KRT18; both Santa Cruz, Dallas, TX, http://www.scbt.com), KDR (EMD Millipore, Billerica, MA, http://www.emdmillipore.com). Slides were mounted in Mowiol medium (EMD Millipore). Fluorescence imaging was performed on a Leica DMI4000B automated inverted microscope (Leica Microsystems, Buffalo Grove, IL, http://www.leica‐microsystems.com), and confocal fluorescence imaging was performed on an Olympus IX83 (Olympus, Tokyo, Japan, http://www.olympus‐lifescience.com) equipped with FV10‐ASW 4.2 software. Incubation with secondary antibodies alone did not give any detectable background signal. Percentage of positive cells for ki67, Oct4, and TTF1was calculated by random field count of positive cells normalized to the number of total nuclei in the field.

### Conditioned Media Screening

After 5 days of culture on poly‐d‐lysine in PGM and after thorough PBS washing, serum‐free CEM medium was conditioned for 24 hours by PSs and thus contained only proteins secreted by the cells. Serum‐free CEM was also conditioned by PDCs in an equivalent ratio of cell number to media volume. Medium was centrifuged at 2,000 rcf for 5 minutes and then stored at −80°C until analysis. Media were analyzed by membrane‐based enzyme‐linked immunosorbent assay (RayBio Human Cytokine Antibody Array 5; Ray Biotech, Norcross GA, http://www.raybiotech.com), according to the manufacturer’s instructions. Densitometric analysis was performed by ImageJ software, and data were presented either as optical density values or PS‐to‐PDC ratio.

### Statistical Analysis

All results are presented as mean value ± SEM unless otherwise specified. Significance of difference between any two groups was determined by two‐sided Student *t* test, and a final value of *p* < .05 was considered significant.

## Results

We tested at first how different substrates—GEL, FN, and LAM—affected the efficiency of the protocol to isolate LPCs as spheroids, namely PSs and as PDCs. We also performed preliminary optimization of cell culture media: F12 and DMEM low‐glucose media could not successfully promote explant outgrowth (data not shown), whereas CEM on each coating yielded sufficient outgrowth EDCs after 2–3 weeks (Fig. 1B). A significant subset of EDCs plated on poly‐d‐lysine formed PSs, consistently with a recognized stemness feature [32], whose yield and size were assessed after 1 week and were independent of the explant coating (Fig. 1C, 1D).

**Figure 2:**
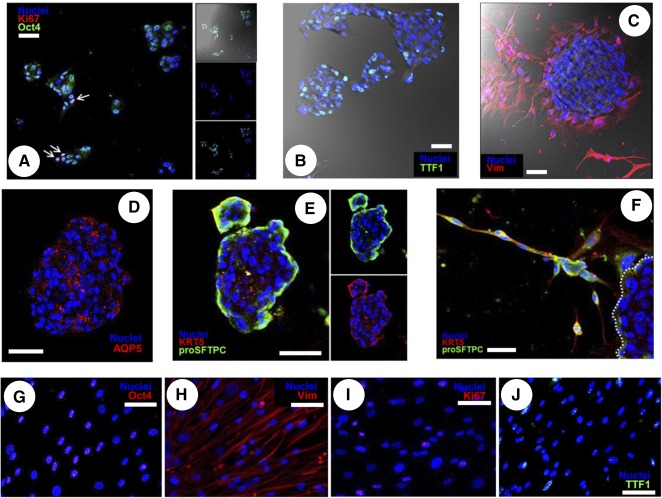
Protein expression profile of pneumospheres (PSs) and pneumosphere‐derived cells (PDCs) in different culture conditions by immunofluorescence. **(A–E):** Representative confocal images of PSs. **(A):** Small, early‐forming PSs were highly positive for the stemness marker Oct4 and the proliferation marker ki67, with frequent double‐stained cells (white arrows). **(B):** Mature PSs remained positive for the transcription factor TTF‐1. **(C):** Few cells inside the PS were positive to VIM staining, particularly on the outer layers, but cells migrating out of the PS started expressing VIM as they attached as monolayers. **(D):** Aquaporin 5 was expressed by a scattered portion of cells inside the PS. **(E):** Cells on the outer layers of the PS were strongly positive to KRT5 and pro‐SFTPC staining, consistent with a phenotypical gradient. **(F):** Examples of clusters of KRT5+/SFTPC+ double‐positive cells just detached from a PS (dotted line) could be identified. Representative fluorescence images of PDCs are shown, which remained highly positive only for Oct4 **(G)**, Vim **(H)**, ki67 **(I)**, and TTF‐1 **(J)** staining. All PS images were taken at the confocal plane of the core unless otherwise specified. Scale bars = 50 µm. Abbreviation: Vim, vimentin.

PSs were analyzed by immunofluorescence staining and confocal microscopy, and no differences were observed in protein abundance or distribution among PSs derived from different coatings (data not shown). Representative panels for PS staining are shown in Figure 2; their features were comparable to other described spheroid models. In fact, small PSs at an early stage of growth were highly positive for the stemness marker Oct4 [33], and proliferating double‐positive Ki67+/Oct4+ cells could be identified (Fig. 2A). Also, PSs contained a majority of TTF1+ cells (Fig. 2B) while expressing very low levels of VIM. When attaching to the culture plate surface, however, PDCs spreading from the PS as a monolayer were highly positive to vimentin staining (Fig. 2C), suggesting the acquisition of mesenchymal traits and cytoskeletal remodeling. PSs contain some dispersed cells positive for AQP5 (Fig. 2D), whereas KRT5 and pro‐SFTPC were detectable in the outer layers (Fig. 2E), consistently with a commitment gradient inside the spheroid. At early timing of attachment, examples of clusters of cells migrating from the PSs and still highly positive for KRT5 and pro‐SFTPC could be detected (Fig. 2F). Cells positive for vascular endothelial growth factor receptor 2 (KDR) and cytokeratin 18 (KRT18) were not detectable at this stage in any of the coatings tested (data not shown). Once fully attached and spread in 2D, relevant subsets of PDCs expressed ki67 (15.8% ± 1.5%), Oct4 (45.2% ± 4.6%), and thyroid transcription factor‐1 (TTF1; 38.6% ± 2.9%) independently of the coating, as assessed by quantification of immunofluorescent staining; most were positive for vimentin (Fig. 2G–2J).

**Figure 3:**
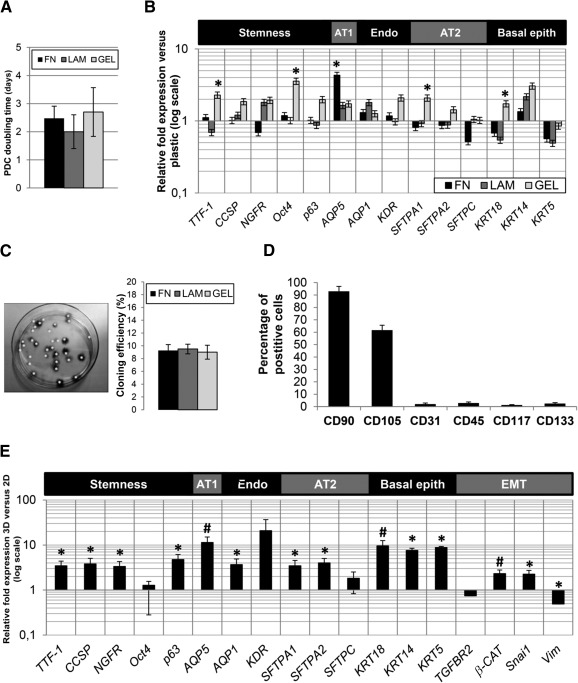
PDC phenotype profiling and comparative gene expression profile with pneumospheres (PSs). **(A):** PDC doubling times on different substrates. **(B):** Gene expression levels for multiple markers of interest were analyzed by real‐time polymerase chain reaction (PCR) and compared among different coatings. **(C):** Representative image of PDC clonogenesis experiments, and corresponding efficiency analysis of different coatings by semiautomatic identification and quantification of clones (white asterisks). **(D):** PDC immunophenotype was unaffected by the coating and was analyzed by cytofluorimetry for a panel of hematopoietic, vascular, and mesenchymal markers, supporting their stromal nature. **(E):** Gene expression levels were analyzed by real‐time PCR and compared between PSs and PDCs. Expression levels for genes of both undifferentiated and committed profiles were significantly upregulated in three‐dimensional (3D) PSs compared with two‐dimensional (2D) PDCs, suggesting an enhanced niche‐like microenvironment inside the PS. ∗, *p* < .05 versus plastic or 2D; #, *p* < .01 versus 2D. Abbreviations: AT1, alveolar type 1 pneumocyte; AT2, alveolar type 2 pneumocyte; EMT, epithelial to mesenchymal transition; Endo, endothelium; epith, epithelium; FN, fibronectin; GEL, gelatin; LAM, laminin.

PDC monolayers were highly proliferating and undifferentiated, with comparable doubling times between 2 and 2.5 days on different coatings (Fig. 3A). Comparative expression analysis by real‐time PCR for a panel of genes of interest (markers of stemness, pneumocytes, endothelium, and epithelium) in PDCs (Fig. 3B) revealed that for a subset of genes (*TTF1*, *Oct4*, *SFTPA1*, *KRT18*), gelatin coating was associated with slightly, albeit significantly, higher levels than fibronectin and laminin, with other genes (i.e., *CCSP*, *p63*) showing similar biological trends without reaching statistical significance. PDCs on fibronectin coating had significantly higher RNA levels of *AQP5*, with a clear trend toward downregulation for *SFTPC*, suggesting a preferential commitment toward an alveolar type 1 (AT1) phenotype.

PDCs contained a subpopulation with clonal growth capacity, yielding a comparable cloning efficiency of approximately 9% on all coatings (Fig. 3C). The PDC immunophenotype was consistent with previously described results, and was unaffected by coating, with approximately 95% CD90+ and significant subpopulations of CD105+ cells (Fig. 3D) but negative or very low CD45, CD31, CD117, and CD133 subpopulations; this was consistent with a nonhematopoietic and nonendothelial lineage.

Because culture coatings seemed to have a negligible effect on the phenotype of our cell model, we next investigated the influence of the spontaneous 3D‐culture switch by comparing gene expression profiles of PSs, normalizing to corresponding PDCs for each coating (Fig. 3E). Irrespective of the coating, almost all markers were significantly upregulated in PSs compared with PDCs, considering a wide panel of genes for stemness (*TTF‐1*, *CCSP*, *NGFR*, and *p63*), pneumocytes (i.e., *AQP5*, *SFTPA1*, and *SFTPA2*), endothelium (i.e., *AQP1* and *KDR*), and basal epithelium (i.e., *KRT18*, *KRT14*, and *KRT5*). This suggested a spontaneous 3D organization in a microtissue‐like structure, inducing epithelial committed phenotypes. The significant, albeit slight, upregulation of *Snai1* and β‐catenin also suggested that partial EMT mechanisms are involved in PS formation, as already described for other spheroid culture systems [9, 34, 35, 36]. Vimentin expression was significantly downregulated in PSs versus PDCs, consistently with the observed immunofluorescence staining (Fig. 2C, 2H). Markers of lung endothelial phenotype (i.e., *AQP1*, *KDR*) were upregulated, although endothelial proteins were never detectable by immunofluorescence (data not shown) and cytofluorimetry (Fig. 3D).

PDCs maintained sphere‐forming capacity, because they were able to yield IIPSs once they were plated back in the appropriate conditions. IIPSs resembled all features of primary PSs. In fact, a clear on/off switch in the expression of key epithelial markers (KRT5, pro‐SFTPC) could be observed between 3D (both PSs, IIPSs) and 2D (PDCs) culture by immunofluorescence staining, with the opposite switch for vimentin expression (supplemental online Fig. 1). To further characterize the phenotype, we performed a double‐labeling EpCam/CD90 for flow cytometry. Cells were mainly CD90+ at all stages (supplemental online Fig. 2a). Results showed that a small EpCam+/CD90+ double‐positive population was present in a constant proportion among EDCs, PSs, PDCs, and secondary PSs stages (supplemental online Fig. 2b–2d), thus suggesting the stromal, albeit plastic, nature of the cell population overall.

To fully elucidate the apparent mesenchymal traits acquired in 2D culture by PDCs, we also performed a mesodermal multilineage differentiation assay on EDCs and PDCs for the three mesenchymal lineages: osteogenic, chondrogenic, and adipogenic (supplemental online Fig. 3). EDCs displayed an incomplete mesenchymal potential, because osteogenic differentiation was not detectable. PDCs selected after the spheroid stage, however, contained a subpopulation with partial ability for multilineage mesodermal transdifferentiation. Nonetheless, such potential was dramatically lower than that of lung mesenchymal stem cells (L‐MSCs), independently isolated from lung parenchyma, shown as the positive control in supplemental online Figure 2. Thus, PDCs significantly differed from resident L‐MSCs, and the mesenchymal‐like traits acquired in 2D seemed consistent with a transient mesenchymal transition of progenitors induced when not in optimal 3D culture conditions.

**Figure 4:**
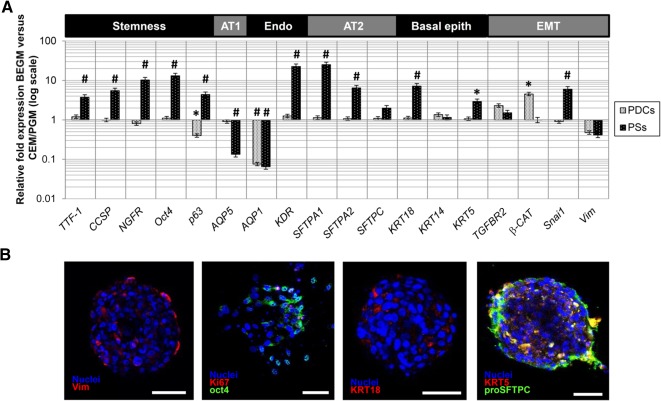
Differential response of PSs and PDCs to BEGM culture. **(A):** PSs were responsive to 1 week of culture in BEGM medium, as shown by significant upregulation of multiple gene expression levels compared with control medium, whereas PDCs were mostly unresponsive. The *y*‐axes are plotted on the log scale. **(B):** As indicated by immunofluorescence staining after BEGM culture, vimentin expression in PSs was low under control conditions, and high expression levels of Oct4 were confirmed inside the PS. KRT18 expression was low but detectable, whereas pro‐SFTPC was expressed in outer cell layers on a thicker portion than under basic culture conditions. All PS images were taken at the confocal plane of the core, unless specified. Scale bars = 50 µm. ∗, *p* < .05; #, *p* < .01 versus the reference (two‐dimensional culture or control medium). Abbreviations: AT1, alveolar type 1 pneumocyte; AT2, alveolar type 2 pneumocyte; BEGM, bronchial/epithelial growth medium; CEM/PGM, complete explant medium/pneumosphere growth medium; EMT, epithelial to mesenchymal; Endo, endothelium; epith, epithelium; PDC, pneumosphere‐derived cell; PS, pneumosphere.

Next, we performed a differential gene expression analysis between PSs and PDCs after 1 week of culture in BEGM media (Fig. 4A) to test responsiveness to definite pulmonary commitment. PSs were strikingly more responsive to the specific stimuli than corresponding PDCs, indicating both maintenance and commitment of undifferentiated cells toward epithelial (i.e., AT2, basal cells) phenotypes. In fact, stemness/progenitor genes (i.e., *TTF‐1*, *CCSP*, *NGFR*, *Oct4*, and *p63*), AT2 markers (i.e., SFPTA1 and SFTPA2), and basal epithelial genes (i.e., *KRT18* and *KRT5*) were significantly upregulated compared with control media. AQP5, which was spontaneously upregulated in PSs, was downregulated after BEGM exposure; this was consistent with the induction of a preferential bronchiolar versus alveolar phenotype by this culture media. Endothelial markers were modulated, with a further increase in KDR levels but a decrease in *AQP1*, which was downregulated in a similar proportion in PDCs as well. PDC gene expression levels were mostly unaffected by specific BEGM media treatment except for a slight decrease in *p63* expression and increase in β‐CAT expression. PSs cultured in BEGM were also analyzed by immunofluorescence and confocal microscopy (Fig. 4B). Vimentin expression was confirmed to be very low, whereas strong Oct4 expression was detectable. Few KRT18+ cells were detectable inside PSs after BEGM culture; KRT5+ and SFPTC+ cells were still detectable in outer PS layers but appeared to involve a thicker portion than basic culture conditions, consistent with the upregulated gene expression levels.

**Figure 5:**
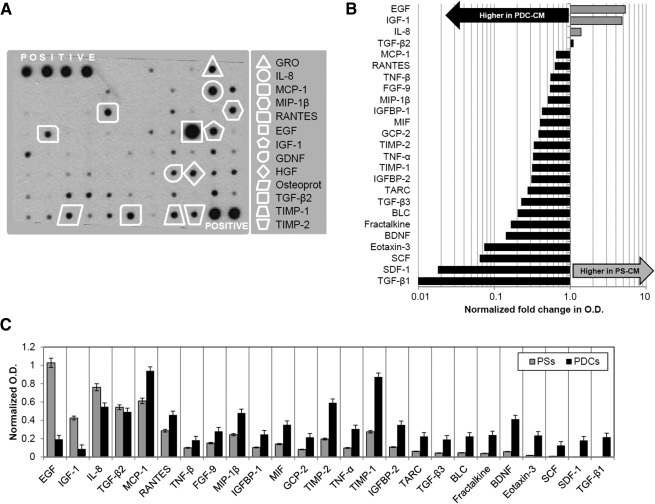
PS‐CM and PDC‐CM screening for paracrine and signaling molecules. **(A):** Representative profile of PS‐CM analyzed by protein array. A selection of growth factors, chemokines, and cytokines of interest is highlighted in the panel. A complete list of factors screened by the assay is presented in supplemental online Table 2. Differential analysis of conditioned media from PSs and PDCs revealed a distinctive modulation in the secretion profile of a subset of cytokines. PS/PDC ratios are plotted in **(B)**, direct level comparison is shown in **(C)**. Factors in plots are listed based on decreasing PS/PDC ratio. Abbreviations: O.D., optical density; PDC, pneumosphere‐derived cell; PDC‐CM, pneumosphere‐derived cell‐conditioned medium; PS, pneumosphere; PS‐CM, pneumosphere‐conditioned medium.

Cellular interactions inside a stem cell niche are regulated by both direct contacts and paracrine signaling; therefore, we performed PS‐conditioned media (PS‐CM) screening to define their specific secretomic profile compared with monolayer PDCs. PSs produce and release a wide panel of humoral factors, some of which are already described [14], including cytokines, chemokines, growth factors, and regulatory proteins (Fig. 5A; supplemental online Table 2). In particular, the strongest array signal was that of EGF, together with highly detectable levels of other growth factors such as insulin‐like growth factor‐1 (IGF‐1), hepatocyte growth factor (HGF), and glial cell‐derived neurotrophic factor (GDNF). Binding proteins, such as insulin‐like growth factor‐binding protein (IGFBPs), that regulate growth factor availability and are suggested to play roles in stem cell biology [37, 38], were produced as well. PSs secreted chemokines, such as IL‐8, MCP‐1, MIP‐1α, and RANTES; and proteins, such as osteoprotegerin, with a well‐known immunomodulatory function. Metalloprotease inhibitors (i.e., TIMP1, TIMP2), mitogenic factors (i.e., GRO) and regulatory factors for EMT and development (i.e., tumor growth factor [TGF]β2, FGF9) [17] were detectable, as well. When comparing the CMs of PSs and PDCs, significantly different secretomic profiles were evident, as shown by plotting optical density ratios of differentially secreted factors (Fig. 5B) or optical densities from the screening of the two CMs (Fig. 5C). Interestingly, the secretion of the epithelial factor EGF had the highest PS/PDC ratio, whereas TGFβ1 the lowest, being secreted only by the more mesenchymal‐like population (i.e., PDCs).

## Discussion

The lung is considered a very plastic organ, harboring multiple cell populations with stemness features, and able to significantly contribute to homeostasis as well as repair and regeneration after injuries [3, 4, 39]. Recently, the isolation of a progenitor population as spontaneous spheroids has been described with therapeutic potential [14]. We have confirmed that sphere formation, here named pneumospheres, can be a reproducible method to isolate LPCs, as also shown in other papers and models [9, 28, 29, 32, 40, 41, 42, 43]. PSs from healthy tissue of young patients represent an ideal model for studying normal stem cell niches ex vivo, personalized disease modeling, screening of differentiation protocols or drugs effects, ex vivo lung bioengineering protocols, and possible cell collection from optimal allogeneic donors for cell therapy.

We aimed at studying the effects of different culture conditions on gene and protein expression in PSs and PDCs, mirroring the epithelial and mesenchymal plasticity of the organ in vivo [3, 6, 44, 45, 46]. Multiple proteins from the extracellular matrix have been shown to influence cell phenotype and stemness [21, 22]. In our model, different substrates were minimally able to significantly modulate the phenotype of LPCs grown as monolayers of PDCs (Fig. 3B). Conversely, a culture switch to the 3D spheroid system of the PS was very efficient in committing cells toward epithelial phenotypes, independently of the original coating (Figs. 2, 3E; supplemental online Fig. 1). Moreover, LPCs as 3D PSs were significantly more responsive than PDCs to specific commitment stimuli, such as culture in BEGM media (Fig. 4). Overall, these data support the creation of a 3D, proepithelial niche‐like structure for adult LPCs inside PSs [9, 13].

Results show that PSs and PDCs contain a stromal lung progenitor population with clonal growth capacities (Fig. 3C), together with the expression of TTF‐1, Oct4 [33], pro‐SFTPC, and KRT5 (Fig. 2G–2J), which are markers of AT2 and basal stem cells. Consistently, KRT14 expression, considered as a marker of AT2 regenerative activation after injury [47], was upregulated in PSs. The combination of AT2 and mesenchymal cells has been described as an ex vivo lung niche model [13]. In our study, we further supported this notion by showing, for the first time to our knowledge, the peculiar paracrine properties of human PSs (Fig. 5; supplemental online Table 2). In fact, we have identified several distinctive secreted factors in PS‐CM known to play a key role in lung homeostasis, development, injury, and cell‐cell communications. When comparing the paracrine profile of 3D with 2D culture, a specific modulation is evident of multiple humoral factors (Fig. 5), including a downregulation of proinflammatory cytokines (e.g., MCP1, tumor necrosis factors, GCP2, BLC, eotaxin) and an upregulation of the growth factors EGF and IGF1 in PSs. EGF is the main mitogenic stimuli for AT2, for both normal alveolar renewal and response to injury [48, 49], and it is released in higher amounts in 3D PSs consistently with increased epithelial commitment (Figs. 2, 3E; supplemental online Fig. 1). On the contrary, the mesenchymal factor TGFβ1, which exerts a major role in collagen production and induction of EMT associated with pathological fibrosis [50, 51, 52], is secreted only by PDCs and not in the niche‐like stage of PSs. Although lung progenitor cells derived from spheroids have been shown to exert antifibrotic effects [14], these results suggest that the administration of LPCs as PSs could even enhance this beneficial property. Further studies will clarify this hypothesis.

FGF9 is also produced at higher levels by PSs, in combination with TGFβ2. These factors play key roles in regulating lung development [17], and the powerful combination of FGF signaling and 3D culture on the differentiation efficiency of embryonic stem cells into distal epithelial cells has recently been reported [20]. Interestingly, PSs and PDCs secrete HGF in similar amounts able to sustain clonal self‐renewal and epithelial colony formation, mediating mesenchymal support [54] and epithelial/endothelial crosstalk [55].

Under the conditions of our study, PSs and PDCs contained a majority of CD90+ cells (supplemental online Fig. 2), but we have shown that vimentin positivity marks a significant difference between 3D and 2D culture, suggesting a mesenchymal‐like potential for PDCs, which, nonetheless, do not display a standard lung mesenchymal stem cell phenotype (supplemental online Fig. 1), highlighting the relevant biological and functional differences between PS/PDC and lung MSCs. Thus, PDCs likely represent a single bipotent stromal progenitor population expressing minimal epithelial features but capable of significant epithelial commitment when cultured in optimal spheroid conditions, consistently with similar phenomena observed in vivo [56]. In fact, increased vimentin expression by lung epithelial cells has been described as a TGF‐β1‐dependent reversible response to stress and injury [56], consistently with superior TGF‐β1 release in PDC 2D culture (Fig. 5). These data further confirm how culture as spheroids provides a more physiologically alike and epithelial‐prone microenvironment. Such an environment, in fact, is able to activate the expression of epithelial markers and turn off vimentin.

3D growth has been shown to affect differentiation of bronchospheres [9], and models from multiple tissues have confirmed how EMT can modulate stemness features [35, 36, 57, 58, 59]. In the PS model, multiple lung stemness markers, such as NGFR, Oct4, and p63, were upregulated, both compared with 2D culture (Fig. 3E) and in response to BEGM exposure (Fig. 4). The striking differential and opposite modulation of vimentin levels observed among PSs, PDCs, and secondary PSs (Figs. 2, 3E; supplemental online Fig. 1) further supports loss of mesenchymal traits inside the PS. Also, β‐catenin was significantly upregulated in PSs compared with PDC monolayers, and it has been associated with protection against fibrosis, promotion of epithelial cell survival [60], and prevention of differentiation of epithelial progenitors [61].

## Conclusion

Our data suggest a proepithelial niche‐like microenvironment inside the PS, highly influenced by the 3D culture system and able to modulate cell phenotype. This feature makes PSs more prone to LPC maintenance and more responsive to specific epithelial commitment stimuli than 2D monolayer cell cultures. Also, we have shown for the first time how PSs produce strong paracrine signals and have a distinctive secretomic profile significantly affected by the 3D culture switch. Thus the modulation of 3D growth alone could be a powerful tool to control stemness and epithelial commitment in in vitro models of lung progenitor cells.

## Author Contributions

I.C.: conception and design, collection and/or assembly of data, data analysis and interpretation, manuscript writing, final approval of manuscript; F.P. and G.M.: collection and/or assembly of data, data analysis and interpretation; F.A., C.S., and V.P.: collection and/or assembly of data; E.D.F.: data analysis and interpretation, manuscript writing; M.P. and R.C.: data analysis and interpretation; M.I.: provision of study material; G.B.‐Z.: financial support, data analysis and interpretation; E.M.: conception and design, manuscript writing; G.F.: conception and design, financial support, provision of study material, manuscript writing, final approval of manuscript.

## Disclosure of Potential Conflicts of Interest

The authors indicated no potential conflicts of interest.

## Supporting information

Supporting Information
