# Supplementary material for: Human Lung Spheroids as In Vitro Niches of Lung Progenitor Cells with Distinctive Paracrine and Plasticity Properties
Source: Stem Cells Transl Med. 2016 Sep 22;6(3):767–77. doi: 10.5966/sctm.2015-0374 (PMC5442776; doi:10.5966/sctm.2015-0374)
Supplement: Supplementary file 1 — Supporting Information [file SCT3-6-0767-s001.pdf]

SUPPLEMENTAL INFORMATION – CHIMENTI ET AL.

SUPPLEMENTAL FIGURES.

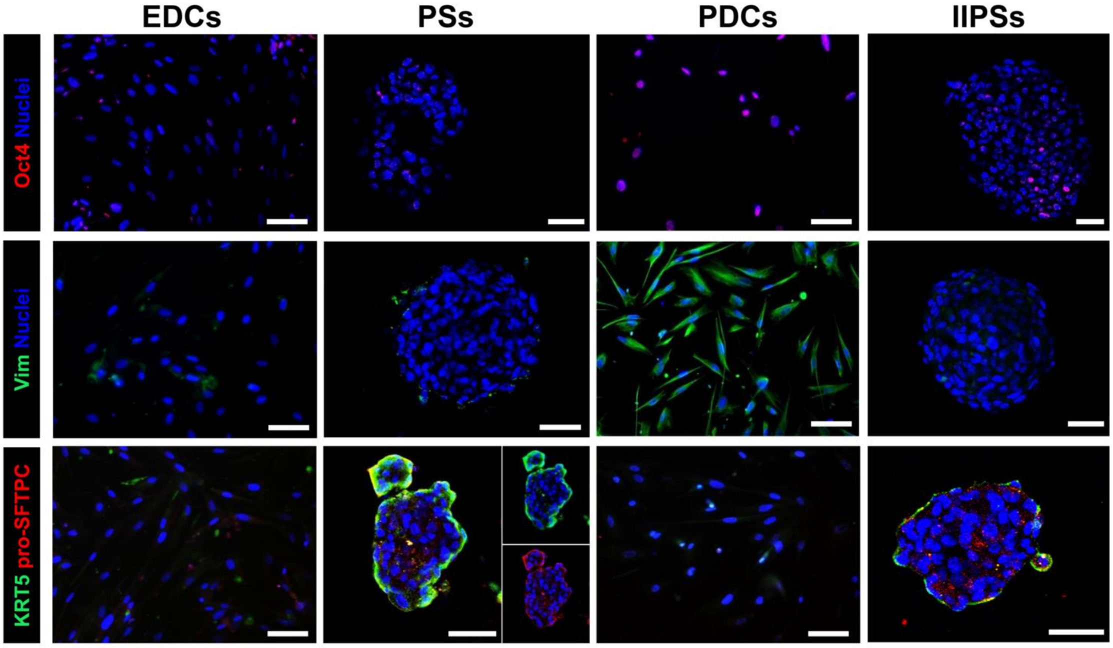

Figure S1. Immunofluorescence stainings at various cell culture stages. Oct4, vimentin (VIM), cytokeratin 5 (KRT5) and pro-surfactant C (pro-SFTPC) stainings at the explant-derived cells (EDCs), pneumospheres (PSs), PS-derived cells (PDCs) and secondary PSs (IIPSs) stages. Oct4+ cells were enriched after the EDC stage, and particularly at the PDC stage. A clear on/off switch was detectable for vimentin (VIM) expression in PDCs, while the opposite regulation was observed for the epithelial markers KRT5 and pro-SFTPC in PSs and IIPSs. Scale bars = 50µm.

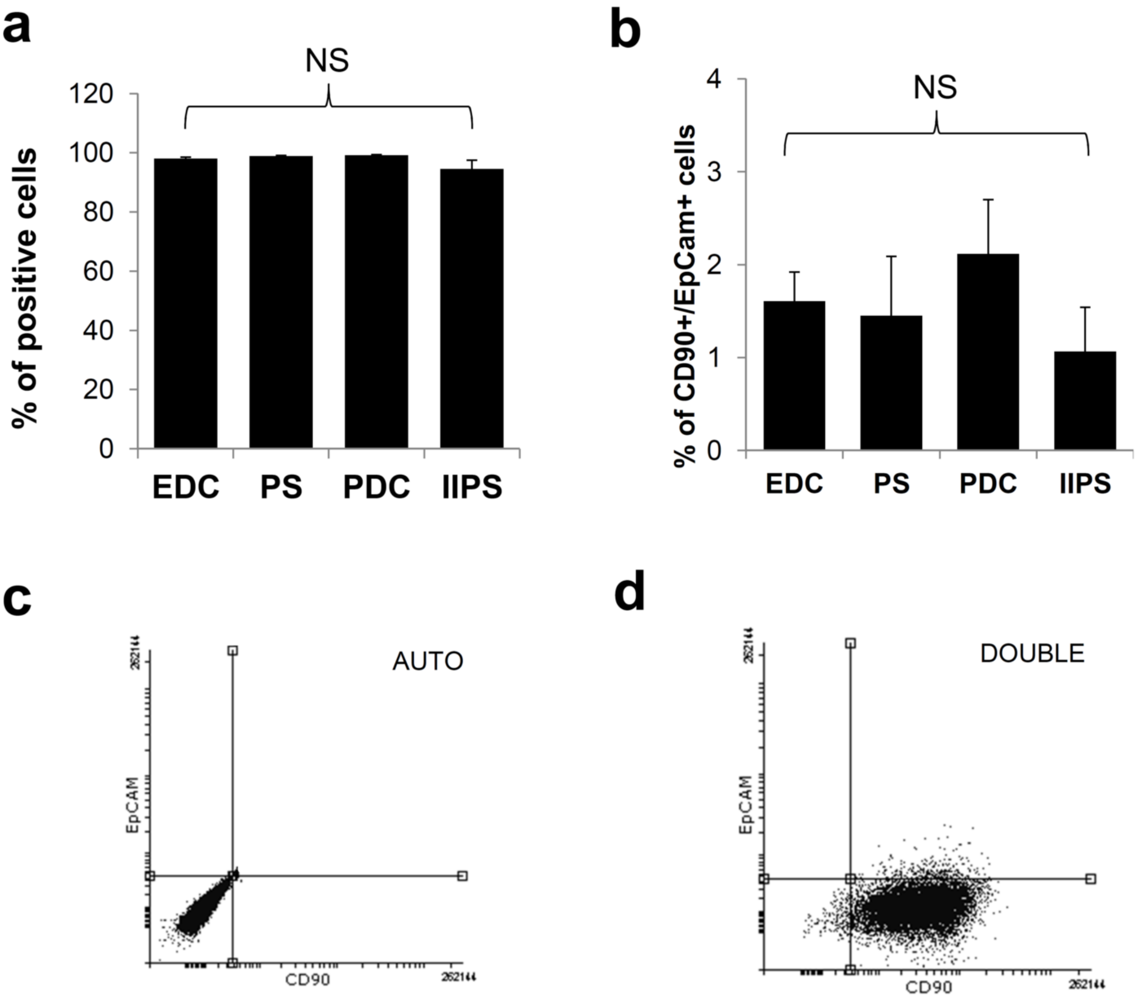

Figure S2. Immunophenotype at different stages of culture. The proportion of CD90+ cells (a) was constant at all culture stages (EDC, PS, PDC, IIPS), as it was the small subpopulation of EpCam+/CD90+ double positive cells. Representative dot-plot panels for the analysis of autofluorescence (c) and double staining (d). NS: not significant.

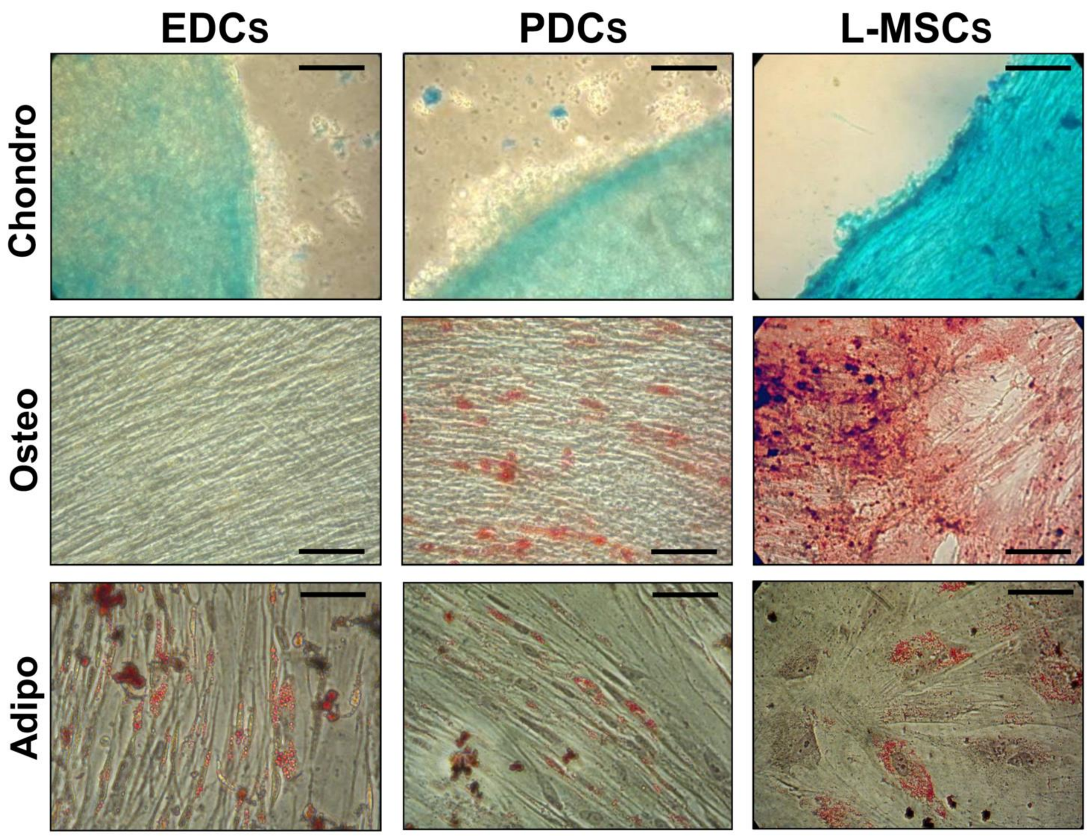

Figure S3. Transdifferentiation assays of EDCs and PDCs for mesenchymal lineages. Chondrogenic, osteogenic and adipogenic transdifferentiation potential of EDCs and PDCs was assessed. The mesenchymal potential of EDCs was not complete, since osteogenic differentiation was not detectable. Conversely, PDCs clearly contained a subpopulation with complete multi-lineage potential, although such potential was dramatically lower than pure lung mesenchymal stem cells (L-MSCs) isolated from lung parenchyma. Scale bars = 50µm.

SUPPLEMENTAL TABLES

Table S1. Primers sequence for realtime PCR.

Target	Sequence	Tm (°C)	Size[bp]	Other names
**TTF-1 fw**	ACCAGGACACCATGAGGAAC	59,8	116	Nkx2.1
**TTF-1 rv**	GCTCATGTTCATGCCGCT	60,9		
**SFTPA1 fw**	ATCCCTGGAGAGTGTGGAGA	59,6	102	
**SFTPA1 rv**	AAAGTCGTGGAGTGTGGCTT	59,7		
**SFTPA2 fw**	CTGGAGAGCGTGGAGAGAAG	60,2	98	
**SFTPA2 rv**	GAAGTCGTGGAGTGTGGCTT	60,3		
**SFTPC fw**	AGCAAAGAGGTCCTGATGGA	59,8	103	
**SFTPC rv**	CGATAAGAAGGCGTTTCAGG	59,8		
**SCGB1A1 fw**	ATGAAACTCGCTGTCACCCT	59,7	114	CCSP
**SCGB1A1 rv**	TGTGTCCATGAGGAGGGTTT	60,3		
**TP73L fw**	CCACCCTACAGTACTGCCCT	59,1	114	p63
**TP73L rv**	TTGTCTGTGTGCTCTGGGAC	59,9		
**NGFR fw**	CTGCTGCTGTTGCTGCTTCT	62	98	p75
**NGFR rv**	CAGGCTTTGCAGCACTCAC	60,7		
**KRT5 fw**	GGAGCTCATGAACACCAAGC	60,8	104	CK5
**KRT5 rv**	CTGGTCCAACTCCTTCTCCA	60,2		

**KRT14 fw**	GGCCTGCTGAGATCAAAGAC	60	105	CK14
**KRT14 rv**	TCTGCAGAAGGACATTGGC	60		
**KRT18 fw**	GAGCACTTGGAGAAGAAGGG	59	126	CK18
**KRT18 rv**	GTCAATCTGCAGAACGATGC	59,4		
**AQP1 fw**	CTCTCAGGCATCACCTCCTC	59,9	109	
**AQP1 rv**	GGAGGGTCCCGATGATCT	59,8		
**AQP5 fw**	TGGCATCCTCTACGGTGTG	60,6	119	
**AQP5 rv**	GCTGGAAGGTCAGAATCAGC	59,9		
**Oct4 fw**	GTGGAGGAAGCTGACAACAA	58,9	118	
**Oct4 rv**	TCTCCAGGTTGCCTCTCACT	60		
**TGFBR2 fw**	CTGCACATCGTCCTGTGG	59,8	110	
**TGFBR2 rv**	GGAAACTTGACTGCACCGTT	60,1		
**CTNNB1 fw**	AGGTCTGAGGAGCAGCTTCA	60,2	142	beta-catenin
**CTNNB1 rv**	ATTGTCCACGCTGGATTTTC	59,9		
**Snai1 fw**	CTTCTCTAGGCCCTGGCTG	59	134	
**Snai1 rv**	CATCTGAGTGGGTCTGGAGG	59		
**Vim fw**	ACCCACTCAAAAAGGACACTTC	59	88	
**Vim rv**	GGTCATCGTGATGCTGAGAA	59		

Tm: melting temperature.

Table S2. Complete panel of proteins screened by protein array in conditioned media.

POS	POS	POS	POS	NEG	NEG	ENA-78	GCSF	GM-CSF	GRO	GRO-α
I-309	IL-1α	IL-1β	IL-2	IL-3	IL-4	IL-5	IL-6	IL-7	IL-8	IL-10
IL-12	IL-13	IL-15	IFN-γ	MCP-1	MCP-2	MCP-3	MCSF	MDC	MIG	MIP-1β
MIP-1δ	RANTES	SCF	SDF-1	TARC	TGF-β1	TNF-α	TNF-β	EGF	IGF-1	Angiogenin
Oncostati M	Thrombopoietin	VEGF	PDGF-BB	Leptin	BDNF	BLC	CCL3	Eotaxin	Eotaxin-2	Eotaxin-3
FGF-4	FGF-6	FGF-7	FGF-9	Flt-3 ligand	Fractalkine	GCP-2	GDNF	HGF	IGFBP-1	IGFBP-2
IGFBP-3	IGFBP-4	IL-16	IP-10	LIF	LIGHT	MCP-4	MIF	MIP-3α	NAP-2	NT-3
NT-4	Osteopontin	Osteoprotegerin	PARC	PLGF	TGF-β2	TGF-β3	TIMP-1	TIMP-2	POS	POS
